# Clinical epigenetics settings for cancer and cardiovascular diseases: real-life applications of network medicine at the bedside

**DOI:** 10.1186/s13148-021-01047-z

**Published:** 2021-03-30

**Authors:** Federica Sarno, Giuditta Benincasa, Markus List, Albert-Lazlo Barabasi, Jan Baumbach, Fortunato Ciardiello, Sebastiano Filetti, Kimberly Glass, Joseph Loscalzo, Cinzia Marchese, Bradley A. Maron, Paola Paci, Paolo Parini, Enrico Petrillo, Edwin K. Silverman, Antonella Verrienti, Lucia Altucci, Claudio Napoli, the International Network Medicine Consortium, Federica Sarno, Giuditta Benincasa, Markus List, Albert‑Lazlo Barabasi, Jan Baumbach, Fortunato Ciardiello, Sebastiano Filetti, Kimberly Glass, Joseph Loscalzo, Cinzia Marchese, Bradley A. Maron, Paola Paci, Paolo Parini, Enrico Petrillo, Edwin K. Silverman, Antonella Verrienti, Lucia Altucci, Claudio Napoli

**Affiliations:** 1 Department of Precision Medicine, University of Campania “Luigi Vanvitelli”, Napoli, Italy; 2 Department of Advanced Medical and Surgical Sciences (DAMSS), University of Campania “Luigi Vanvitelli”, Naples, Italy; 3 Chair of Experimental Bioinformatics, TUM School of Life Sciences Weihenstephan, Technical University of Munich, Freising, Germany; 4 Network Science Institute and Department of Physics, Northeastern University, Boston, MA USA; 5 Channing Division of Network Medicine, Brigham and Women’s Hospital, Harvard Medical School, Boston, MA USA; 6 Department of Network and Data Science, Central European University, Budapest, Hungary; 7 Department of Mathematics and Computer Science, University of Southern Denmark, Odense, Denmark; 8 Chair of Computational Systems Biology, University of Hamburg, Notkestrasse 9, Hamburg, Germany; 9 School of Health, Unitelma Sapienza University, Rome, Italy; 10 Department of Computer, Control, and Management Engineering, Sapienza University, Rome, Italy; 11 Department of Translational and Precision Medicine, Sapienza University, Rome, Italy; 12 Department of Experimental Medicine, Sapienza University of Rome, Rome, Italy; 13 Department of Medicine, Brigham and Women’s Hospital, Harvard Medical School, Boston, MA USA; 14 Department of Laboratory Medicine and Department of Medicine, Karolinska Institute and Karolinska University Hospital, Stockholm, Sweden; 15 Department of General Internal Medicine and Primary Care, Brigham and Women’s Hospital, Boston, MA USA; 16 Clinical Department of Internal Medicine and Specialistic Units, AOU, University of Campania “Luigi Vanvitelli”, Naples, Italy

**Keywords:** Epigenetics, Cancer, CVD, Precision medicine, Network medicine, Algorithms, Epi-drugs

## Abstract

Despite impressive efforts invested in epigenetic research in the last 50 years, clinical applications are still lacking. Only a few university hospital centers currently use epigenetic biomarkers at the bedside. Moreover, the overall concept of precision medicine is not widely recognized in routine medical practice and the reductionist approach remains predominant in treating patients affected by major diseases such as cancer and cardiovascular diseases. By its’ very nature, epigenetics is integrative of genetic networks. The study of epigenetic biomarkers has led to the identification of numerous drugs with an increasingly significant role in clinical therapy especially of cancer patients. Here, we provide an overview of clinical epigenetics within the context of network analysis. We illustrate achievements to date and discuss how we can move from traditional medicine into the era of network medicine (NM), where pathway-informed molecular diagnostics will allow treatment selection following the paradigm of precision medicine.

## Introduction

Despite advances in early detection, therapeutic strategies, and supportive care, cancer and cardiovascular diseases (CVDs) remain the leading causes of morbidity and mortality worldwide [6, 140], WHO reveals leading causes of death and disability worldwide: 2000–2019, Cancer (who.int)). In 2015, then US President Barak Obama launched a precision medicine initiative [105] with the goal of refining the current standard of care. To achieve this goal, noninvasive biomarkers are needed that can diagnose diseases on a mechanistic level, as well as novel or repurposed drugs that are able to target precisely these mechanisms. Integration of clinical with genomics or even multi-omics datasets such as transcriptomics and proteomics [146] has revealed novel disease-specific molecular pathways in cancer [12, 43, 102] and CVDs [76]. However, the connection of hereditary genetic determinants (which may allow patient stratification) and the individual risk for cancer or CVDs is lacking. This "missing heritability" eludes us due to the complex interplay of multiple factors in complex diseases, including epigenetics, which has already been shown to provide new insights into oncogenesis [30, 31, 45, 102] and CVD [98–101]. Epigenetic modifications may bridge the gap between the genome and the environment revealing early signs of disease onset even in the early phase of fetal development, where epigenetic factors have been associated with an increased risk of CVDs in later life (transgenerational effect) [36, 98–101].

Epigenetics comprises regulatory mechanisms that do not affect the DNA sequence but alter chromatin compaction to modulate gene expression [46]. DNA methylation, regulation of chromatin accessibility, and histone tail modifications are key moderators of vital cellular processes, such as differentiation, survival, and response to external stimuli [46]. These mechanisms individually or in combination are often related to pathogenesis and offer an opportunity to improve disease diagnosis and to predict clinical outcomes [17]. As epigenetic modifications are partially reversible, chromatin-acting epi-drugs can be used to treat complex human diseases. DNA methyl transferase inhibitors (DNMTi), histone deacetylase inhibitors (HDACi), and enhancer of zeste 2 polycomb repressive complex 2 subunit inhibitors (EZH2i) have rapidly reached considerable clinical relevance. For the treatment of hematologic malignancies, one epigenetic biomarker [124] and eight epi-drugs Table 1 have been approved by the FDA and are currently in clinical trials for different solid malignancies Table 2. In contrast, there are, as yet, no approved epi-drugs for CVDs, however, more than twenty Phase 3–4 randomized trials are currently ongoing to evaluate the epigenetic efficacy of the so-called “repurposed” drugs against CVDs, including metformin, statins, and apabetalone, a bromodomain inhibitor with quinazoline structure [98, 99] Table 2.

**Table 1:** FDA approved epi-drug for cancer treatment

Epi-drug	Development Overview	Characteristics	Mechanism of Action	Data of approval	Disease approval	Phase 3	Phase 2	Phase 1/2	Most Recent Events
Vidaza	Clinical studies began in 1980s. However, in the first studies its activity had not been maximized as the mechanism had not yet been identified. In 2004 Azacitidine was marketed as Vidaza in the USA, in subcutaneous administration, for the treatment of five subtypes of MDS. In January 2007, the FDA approved the administration for intravenous use. Azacitidine is available in the EU and several Asia–Pacific countries for the treatment of high-risk MDS, AML, and CML, in Canada and Japan for MDS high risk and a subgroup of AML patients who are not eligible for stem cell transplantation. In September 2020 the drug was approved in oral administration (ONUREG), for AML patients in first complete remission or with complete remission but incomplete blood recovery after chemotherapy (NCT0175735)	Antineoplastics; Aza compounds; Pyrimidine nucleosides	Antimetabolites; DNMT inhibitors	2004 for MDS 2020 for AML	MDS, AML and CML	TL	BC; Leukemia; NC; NSCLC; Peripheral TL	CC; Diffuse large B cell lymphoma; RC; Solid tumors	*25 May 2020* Preregistration for AML (First-line therapy) in European Union (PO)
Decitabine	Decitabine is developed by Otsuka and Janssen-Cilag for MDS and AML. The compound is used in the USA and many other countries outside the EU for MDS and in Malaysia for CML and AML in the EU. The product is also undergoing regulatory review in China for MDS and AML. Clinical development is ongoing for MDS and ovarian cancer. Decitabine has shown limited efficacy against solid tumors. A combined, orally bioavailable, fixed-dose formulation of decitabine plus cedazuridine was approved in 2020 for MDS	Antineoplastics; Aza compounds; Deoxyribonucleosides; Pyrimidine nucleosides	DNMT inhibitors	2006 for MDS	MDS, AML and CML	T-ALL, T-LBL, T/M-MPAL		Malignant melanoma; OC	*07 Dec 2019* Pharmacodynamics data from a preclinical study in acute myeloid leukemia
Vorinostat	Vorinostat has been launched in the USA, Canada, Japan, Argentina, Chile, and Greece for CTCL treatment. Development of vorinostat was discontinued in mesothelioma non-small cell lung cancer, gynecological cancer, and pancreatic cancer. Vorinostat was in phase II development for colorectal cancer and sickle-cell anemia. However, no development in these indications have been reported	Antineoplastic, hydroxamic acid	HDAC inhibitors	2006	CTLC	MM; MLC	AML; B-cell lymphoma; BC; Glioma; HIV-1; MDS; NHL; ALL	ALL; Lymphoma; Solid tumors	*09 Apr 2019* Merck & Co completes a phase II trial in B-cell lymphoma in South-East Asia (PO) (NCT00875056)
Romidepsin	Romidepsin developed by Celgene was discovered by Fujisawa Pharmaceutical from Chromobacterium violaceum. The intravenous formulation of romidepsin was tested in the USA for CTLC, PTCL. Clinical development is underway for breast cancer, multiple myeloma, T-cell leukemia, and T-cell lymphoma in several countries. In 2015 Romidepsin was in pre-registration phase for peripheral T-cell lymphoma and in phase II for cutaneous T-cell lymphoma, and studies for the treatment of renal cell carcinoma, prostate cancer, and pancreatic cancer were stopped	Antineoplastics; Cytostatic antibiotics; Depsipeptides	HDAC inhibitors	2009	CTLC, PTCL			BC; MM; T-LL	*11 Apr 2020* Celgene Corporation and University of California completes a phase I trial in Cutaneous T-cell lymphoma in the USA (NCT01902225) *25 Mar 2020* Celgene Corporation suspends a phase-I/II clinical trials in breast cancer (combination therapy) in the USA, due to five patients’ responses pending (NCT02393794)
Belinostat	Belinostat is developed by Onxeo and Spectrum Pharmaceuticals. Intravenous belinostat is available in the USA and is under regulatory review in Canada as a monotherapy for PTCL. Clinical trial is ongoing for B-cell lymphoma and glioblastoma, in the USA, and for NSCLC in the USA and Denmark. Development for the treatment of multiple myeloma has been stopped	Acrylamides; Antineoplastics; Hydroxamic acids	HDAC inhibitors	2014	PTCL		B-cell lymphoma; Glioblastoma	NSCLC	*10 Apr 2020* National Cancer Institute plans a phase II trial for Chondrosarcoma (Combination therapy, second-line therapy or greater) (NCT04340843)
Panobinostat	Panobinostat being developed by Novartis and Secura Bio, for the treatment of cancer and HIV infections, and after approved from the FDA in 2015 for MM	Antineoplastic, hydroxamic acid	HDAC inhibitors	2015	MM	CMML	AML; CLL; CC; Lymphoma; MDS; Myelofibrosis; Neuroendocrine tumors; NHL; T-LBL; RC; TC	BC; HIV-1; Hodgkin’s disease; melanoma	*11 Aug 2020* Phase-I clinical trials in Glioma (In children, In adults, Combination therapy, Second-line therapy or greater) in the USA (PO) (NCT04341311) *22 Jun 2020* Phase I development is ongoing for Myelofibrosis (combination therapy) in Germany, France, UK, Ireland and Italy (NCT01433445)
Ivosidenib	Ivosidenib was approved on July 20, 2018, in oral administration, for adult patients with relapsed or refractory AML with IDH1 mutation. From May 2019, the drug was approved also for newly diagnosed AML with a susceptible IDH1 mutation in patients who are at least 75 years old or that cannot be treated with chemotherapy (NCT0274839)	Antineoplastics; Cyclobutanes; Pyridines; Pyrrolidines	IDH1 inhibitors	2018	AML with IDH1 mutation	Cholangiocarcinoma; MDS			*30 Jul 2020* Agios pharmaceuticals announces to submit sNDA to US FDA for Cholangiocarcinoma, in second-line therapy in the 2021
Enasidenib	In August 2017, Enasidenib (IDHIFA®), produced by Celgene, was approved from the FDA for the treatment of adult patients with relapsed or refractory AML with IDH2 mutation. The drug is used first for AML in the USA and then approved in Australia. Clinical developments are ongoing for AML, solid tumors, CML, and MDS Other potential indications include a genetic neurometabolic disorder (Type II D-2-HGA; 2-Hydroxyglutaricaciduria)	Amines; Antineoplastics; Pyridines; Triazines	IDH2 inhibitors	2017	AML with IDH2 mutation	MDS		Solid tumor	*12 Jun 2020* Celgene reinitiates a phase II trial in adult patients AML as second-line therapy in the USA (PO) (NCT03881735)
Tazemetostat	Tazemetostat, developed by Epizyme in orally administration, is used for MES and FL treatment in the USA. Clinical development of the oral formulation is ongoing for B-cell lymphoma, central nervous system cancer, follicular lymphoma, histiocytosis, mesothelioma, non-Hodgkin’s lymphoma, non-small cell lung cancer, and solid tumors in the USA, Australia, Japan, Canada, and the European Union Eisai and Epizyme with Roche Molecular Systems are developing complementary diagnostics to help companies identify patients with lymphoma with EZH2 gene mutations, to be used in parallel with the clinical development of tazemetostat Clinical development of the suspension formulation of tazemetostat for the treatment of solid tumors was ongoing in the USA. However, as of September 2018, no recent development reports for the formulation have been identified	Antineoplastics; Biphenyl compounds; Dihydropyridines	Inhibitor of wild-type and mutated forms of EZH2 and SMARCA2 and SMARCA4	2020	MES, FL		CNS cancer; DLBCL; Histiocytosis; OC; Mesothelioma; NHL; Peritoneal cancer; Rhabdoid tumor; Solid tumors; Synovial sarcoma; Uterine cancer	Bladder cancer; NSCLC; PC	*08 Jul 2020* Preregistration for FL (Refractory metastatic disease, Second-line therapy or greater) in Japan (PO) *22 Jun 2020* Pharmacodynamics data from a preclinical trial in NSCLC

Abbreviations: MDS: Myelodysplastic Syndrome; CTLC: cutaneous T cell lymphoma; PTCL: peripheral T cell lymphoma; MM: multiple myeloma; MES: metastatic epithelioid sarcoma; AML: Acute Myeloid Leukemia; CMML: Chronic Myelomonocytic Leukemia; MPC: Metastatic Pancreatic Cancer; MLC: Mesothelioma; ALL: acute lymphoblastic leukemia; LLy: acute lymphoblastic lymphoma; T-ALL: Acute T-Lymphocytic Leukemia; T-LBL: T-lymphoblastic leukemia/lymphoma; DLBCL: Diffuse large B-cell lymphoma; NHL: non-Hodgkin lymphoma; BC: Breast cancer; NSCLC: non-small cell lung cancer; CC: Colon cancer; RC: Renal cancer; PC: Prostate cancer; FS: follicular lymphoma; OC: Ovarian cancer; TC: Thyroid cancer; NC: Nasopharyngeal cancer

**Table 2:** Epitherapy in clinical trials for cancer and CVDs

Drug	Conditions	Study type	Study title	Aim	Status/phase	ID
*Drugs in clinical trial*						
SGI-110 (guadecitabine)	AML	302 participants, Interventional, Randomized, Parallel Assignment, Treatment	Phase 3 Randomized, Open-Label Study of Guadecitabine vs Treatment Choice in Previously Treated Acute Myeloid Leukemia	Multicenter, randomized, open-label, parallel-group study of guadecitabine vs treatment choice	Active/Phase 3	NCT02920008
	MDS CMML	408 participants, Interventional, Randomized, Parallel Assignment, Treatment	Guadecitabine (SGI-110) vs Treatment Choice in Adults With MDS or CMML Previously Treated With HMAs	To evaluate the efficacy and safety of guadecitabine in MDS or CMML who failed or relapsed after treatment with azacitidine, decitabine, or both	Active/Phase 3	NCT02907359
	AML	815 participants, Interventional, Randomized, Parallel Assignment, Treatment	SGI-110 in Adults With Untreated Acute Myeloid Leukemia (AML), Not Considered Candidates for Intensive Remission Induction	To compare efficacy and safety between SGI-110 and treatment in adults with previously untreated AML	Completed/ Phase 3	NCT02348489
RX-3117	MPC	46 participants, Interventional, N/A, Single group Assignment, Treatment	RX-3117 in Combination With Abraxane® in Subjects With Metastatic Pancreatic Cancer	To determine the safety profile, dose modification, and pharmacokinetics of oral RX-3117 administered in combination with Abraxane® to subjects with metastatic pancreatic cancer	Completed/ Phase 1/2	NCT03189914
	Solid tumor Metastatic Bladder Cancer	124 participants, Interventional, N/A, Single group Assignment, Treatment	Dose-Finding and Safety Study for Oral Single-Agent to Treat Advanced Malignancies	To determine the maximum tolerated dose in metastatic solid tumors and to estimate anti-tumor activity in subjects with relapsed or refractory pancreatic or advanced bladder cancer	Completed/ Phase 1/2	NCT02030067
INCB059872	Solid tumors Advanced Malignancies Metastatic Cancer	70 participants, Interventional, N/A, Single group Assignment, Treatment	Azacitidine Combined With Pembrolizumab and Epacadostat in Subjects With Advanced Solid Tumors (ECHO-206)	To evaluate the safety and tolerability of INCB059872 with pembrolizumab and epacadostat	Completed Phase 1/2	NCT02959437
	Solid tumors and hematologic malignancy	215 participants, Interventional, N/A, Single group Assignment, Treatment	An Open-Label, Dose-Escalation/Dose-Expansion Safety Study of INCB059872 in Subjects With Advanced Malignancies	To determine the safety, tolerability, efficacy, PK, PD, and the recommended dose(s) of azacitadine and all-trans retinoic acid in AML and in combination with nivolumab in SCLC	Recruiting Phase 1/2	NCT02712905
CI-994	MM	6 participants, Interventional, N/A, Single group Assignment, Treatment	CI-994 in Treating Patients With Advanced Myeloma	To study the effectiveness of CI-994 in treating patients who have advanced myeloma	Completed Phase 2	NCT00005624
	Lung cancer	Interventional, Randomized, Treatment	Gemcitabine With or Without CI-994 in Treating Patients With Advanced Non-small Cell Lung Cancer	To compare the effectiveness of gemcitabine with or without CI-994 in treating patients who have advanced non-small cell lung cancer	Completed Phase 3	NCT00005093
	Pancreatic cancer	Interventional, Randomized, Treatment	Gemcitabine With or Without CI-994 in Treating Patients With Advanced Pancreatic Cancer	To compare the effectiveness of gemcitabine with or without CI-994 in treating patients who have advanced pancreatic cancer	Completed Phase 2	NCT00004861
EPZ-5676 (Pinometostat)	AML with 11q23 rearrangement	36 participants, Interventional, N/A, Single group Assignment, Treatment	Pinometostat and Azacitidine in Treating Patients With Relapsed, Refractory, or Newly Diagnosed Acute Myeloid Leukemia With 11q23 Rearrangement	To study the side effects and the dose of pinometostat with azacitidine in AML with 11q23 rearrangement	Recruiting Phase 1/2	NCT03701295
	AML with 11q23 rearrangement	37 participants, Interventional, N/A, Single group Assignment, Treatment	Pinometostat With Standard Chemotherapy in Treating Patients With Newly Diagnosed Acute Myeloid Leukemia and MLL Gene Rearrangement	To study the side effects and the dose of pinometostat in AML with 11q23 rearrangement	Recruiting Phase 1/2	NCT03724084
TCP	AML	16 participants, Interventional, N/A, Single group Assignment, Treatment	Phase I/II Trial of ATRA and TCP in Patients With Relapsed or Refractory AML and no Intensive Treatment is Possible	To study the feasibility, pharmacodynamics, and effectivity of ATRA and TCP in AML	Phase 1/2	NCT02261779
	AML, MDS	60 participants, Interventional, N/A, Single group Assignment, Treatment	Study of Sensitization of Non-M3 AML Blasts to ATRA by Epigenetic Treatment With Tranylcypromine (TCP)	To study the maximum tolerated and the efficacy dose of TCP with ATRA and Cytarabine	Recruiting Phase 1/2	NCT02717884
CPI-0610	Myelofibrosis AML, MDS/MPD, MDS	271 participants, Interventional, Non-Randomized, Parallel Assignment, Treatment	A Phase 2 Study of CPI-0610 With and Without Ruxolitinib in Patients With Myelofibrosis	Open-label, sequential dose escalation study of CPI-0610 and open-label study of CPI-0610 with and without Ruxolitinib in patients with Myelofibrosis	Recruiting Phase 1/2	NCT02158858
Rocilinostat (ACY-1215)	MM	101 participants, Interventional, Single Group Assignment, Treatment	ACY-1215 (Ricolinostat) in Combination With Pomalidomide and Low-dose Dex in Relapsed-and-Refractory Multiple Myeloma	To evaluate the side effects, determine the best dose and the response rate of ACY-1215 in combination with Pomalidomide and low-dose dexamethasone in patients with relapsed-and-refractory multiple myeloma	Active, not recruiting Phase 1b/2	NCT01997840
Mocetinostat	Urothelial carcinoma	17 participants, Interventional, N/A, Single group Assignment, Treatment	Study of Mocetinostat in Patients With Urothelial Carcinoma Having Inactivating Alterations of Specific Genes	To evaluate whether the number of patients responding to treatment is substantially higher than other available treatments	Completed/ Phase II	NCT02236195
	Hodgkin’s lymphoma	51 participants, Interventional, Non-Randomized, Single group Assignment, Treatment	Study of MGCD0103 (MG-0103) in Patients With Relapsed or Refractory Hodgkin’s Lymphoma	To study the effect of Mocetinostat on patients with relapsed and refractory Hodgkin’s lymphoma	Terminated/ Phase II	NCT00358982
CPI-1205	mCRPC	242 participants, Interventional, Randomized, Parallel Assignment, Treatment	ProSTAR: A Study Evaluating CPI-1205 in Patients With Metastatic Castration Resistant Prostate Cancer	To study the oral administration of CPI-1205 in combination with enzalutamide or abiraterone/prednisone in male patients with mCRPC	Active, not recruiting/ Phase I/II	NCT03480646
	Advanced Solid Tumors	24 participants, Interventional, N/A, Single group Assignment, Treatment	ORIOn-E: A Study Evaluating CPI-1205 in Patients With Advanced Solid Tumors	To determine the maximum tolerated dose and recommended phase 2 dose of CPI-1205 + ipilimumab in patients with advanced solid tumors	Active, not recruiting/ Phase I/II	NCT03525795
*Approved epi-drug ongoing in clinical trial for other treatments*						
Vorinostat	ALL, LLy	1000 participants, Interventional, Randomized, Parallel Assignment, Treatment	Total Therapy XVII for Newly Diagnosed Patients With Acute Lymphoblastic Leukemia and Lymphoma	To study a novel strategies based on inherited and acquired leukemia-specific genomic features and treatment to improve the cure rate and quality of life of ALL and LLy children	Recruiting/ Phase2/3	NCT03117751
	MM	637 participants, Interventional, Randomized, Single group Assignment, Treatment	Study of Vorinostat (MK-0683) an HDAC Inhibitor, or Placebo in Combination with Bortezomib in Patients With Multiple Myeloma (MK-0683-088 AMN)	To study of the efficacy and safety of bortezomib administered in combination with vorinostat in patients with relapsed or refractory MM	Complete/ Phase 3	NCT00773747
	MLC	662 participants, Interventional, Randomized, Single group Assignment, Treatment	Suberoylanilide Hydroxamic Acid (Vorinostat, MK-0683) Versus Placebo in Advanced Malignant Pleural Mesothelioma (0683-014 AM5, EXT1)	To study the safety, tolerability, and anti-tumor effectiveness of vorinostat in oral administration, for MLC	Complete/ Phase 3	NCT00128102
Panobinostat	AML, MDS	350 participants, Interventional, Randomized, Parallel Assignment Treatment	Panobinostat Maintenance After HSCT for High-risk AML and MDS	To compare maintenance treatment with panobinostat versus the standard approach of preemptive DLI alone in patients with poor-risk AML/MDS	Recruiting/ Phase 3	NCT04326764
	PMF, CIM, post-PVMF, PET-MF, SR-GVHD	356 participants, Interventional, Non-Randomized, Parallel Assignment Treatment	CINC424A2X01B Rollover Protocol	To study the efficacy of ruxolitinib and panobinostat in combination for patients that use the study treatment based on the parent protocol	Recruiting/ Phase 4	NCT02386800
Vidaza	AML	488 participants, Interventional, Randomized, Parallel Assignment, Treatment	Study of Vidaza Versus Conventional Care Regimens for the Treatment of Acute Myeloid Leukemia (AML)	To compare the effect of azacitidine to conventional care regimens in AML patients	Complete/ Phase 3	NCT01074047
	AML	47 participants, Interventional, Non-Randomized, Parallel Assignment, Treatment	zacitidine Combined With Homoharringtonie in AML	To validate the efficacy and safety advantages of the regimens that contain homoharringtonie and azacitidine, and to determine which regimen would receive more living benefits in AML	Recruiting/ Phase 3	NCT04248595
	AML, MDS, CMML	187 participants, Interventional, Randomized, Single group Assignment, Treatment	Controlled Study of Post-transplant Azacitidine for Prevention of Acute Myelogenous Leukemia and Myelodysplastic Syndrome Relapse (VZ-AML-PI-0129)	To study if Vidaza will help to control the disease in patients with AML, CMML, or MDS after a stem cell transplant	Complete/ Phase 3	NCT00887068
	AML, MDS, CML	114 participants, Interventional, Randomized, Parallel Assignment, Treatment	Azacytidine + HAG Regimen vs. Azacytidine for Elderly Patients With Newly Diagnosed Myeloid Malignancy	To explore the efficacy and safety of azacytidine and HAG regimen versus azacytidine for elderly patients with Newly Diagnosed MDS/AML/CMML in China	Recruiting/ Phase 4	NCT03873311
Decitabine	T-ALL, T-LBL, T/M-MPAL	100 participants, Interventional, N/A, Single group Assignment Treatment	Study of Decitabine Combined With HAAG Regimen in Newly Diagnosed ETP-ALL/LBL, T/M-MPAL and ALL/LBL With Myeloid or Stem Cell Markers Patients	To evaluate the efficacy and safety of decitabine combined with HAAG	Recruiting/ Phase 3	NCT04446130
	DLBCL	60 participants, Interventional, Randomized, Parallel Assignment Treatment	A Clinical Trial of Decitabine in Relapse and Refractory Diffuse Large B Cell Lymphoma	To evaluate the safety, tolerability, and clinical effects of decitabine with R ± DHAP	Recruiting/ Phase 4	NCT03579082
Tazametostat	Solid tumor	49 participants, Interventional, N/A, Single group Assignment Treatment	Tazemetostat in Treating Patients With Relapsed or Refractory Advanced Solid Tumors, Non-Hodgkin Lymphoma, or Histiocytic Disorders With EZH2, SMARCB1, or SMARCA4 Gene Mutations (A Pediatric MATCH Treatment Trial)	To study the action of tazemetostat in patients with brain tumors, solid tumors, non-Hodgkin lymphoma, or histiocytic disorders that have come back (relapsed) or do not respond to treatment (refractory) and have EZH2, SMARCB1, or SMARCA4 gene mutations	Phase 2	NCT03213665
*Drug repositioning in CVDs*						
Trimetazidine	HFpEF	25 participants, Interventional, Randomized, Placebo-controlled	TrimetaziDine as a Performance-enhancING drug in Heart Failure with Preserved Ejection Fraction	To assess whether trimetazidine improves LV diastolic function by improving myocardial energy metabolism in HFpEF	Ongoing/ Phase 2	EU Clinical Trial Register: 2018-002170-52; NTR registration: NL7830 [van de Bovenkamp AA,
	PAH	26 participants, Interventional, Randomized, Placebo-controlled	The Role of Trimetazidine on Right Ventricle Function in Pulmonary Arterial Hypertension (TRIMETA-PH)	To evaluate the effect of trimetazidine versus placebo in addition to standard PAH regime on right ventricular function	Completed/Phase 2/ 3	NCT03273387
Tocilizumab	PAH	29 participants, Interventional, Single‐arm	A Therapeutic Open Label Study of Tocilizumab in the Treatment of Pulmonary Arterial Hypertension (TRANSFORM-UK)	To assess the safety and efficacy of tocilizumab in PAH	Completed/Phase 3	NCT02676947
Metformin	MetS	40 participants, Interventional, Randomized, Placebo-controlled	Combination of Metformin/Inulin vs Inulin on Adiponectin in Metabolic Syndrome	To compare the effect of the administration of Metformin/agave inulin vs. Agave inulin on adiponectin in patients with MetS	Completed/ Phase 3	NCT02773927
	T2D, HF	30 participants, Observational, Cohort, Prospective	Lipid Accumulation in Heart Transplant From Non-diabetic Donors to Diabetic Recipients (DCM-AHEAD)	To evaluate in the explanted diabetic heart the presence of possible cellular alterations attributable to the diabetic disease discerning from treated and non-treated with metformin	Completed/NA	NCT03546062
	PAH	1899 participants, Observational, Cohort, Prospective	Hormonal, Metabolic, and Signaling Interactions in PAH	To evaluate if an optimal treatment of the dysfunctional metabolic pathways underlying PAH may improve pulmonary vascular function and consequences of the disease	Recruiting/NA	NCT01884051
Apabetalone (RVX 208)	PAH	10 participants, Interventional, Single‐arm, 2‐center study	Apabetalone for Pulmonary Arterial Hypertension: a Pilot Study	To evaluate the possible efficacy of apabetalone in treating PAH	Recruiting/Early Phase 1	NCT03655704
	T2D-CHD	2425 participants, Interventional, Randomized, Placebo-controlled	Effect of RVX000222 on Time to Major Adverse Cardiovascular Events in High-Risk T2DM Subjects With CAD (BETonMACE)	To determine whether RVX000222 in high-risk type 2 diabetes mellitus patients with coronary artery disease increases the time to major adverse cardiovascular events	Phase 3/Active	NCT02586155
n-3 PUFA or Rosuvastatin	HF	6975 participants, Interventional, Randomized, Placebo-controlled	GISSI-HF- Effects of n-3 PUFA and Rosuvastatin on Mortality-Morbidity of Patients With Symptomatic CHF	To demonstrate that, in HF patients treated at the best of recommended therapies, long-term administration of n-3 PUFA or rosuvastatin is more effective than the corresponding placebo	Completed/ Phase 3	NCT00336336 Tavazzi et al. [128]

Abbreviations: AML: Acute Myeloid Leukemia; MDS: Myelodysplastic Syndromes; CMML: Chronic Myelomonocytic Leukemia; MPC: Metastatic Pancreatic Cancer; MLC: Mesothelioma; MDS/MPD: Myelodysplastic–Myeloproliferative neoplasms;mCRPC: Metastatic Castration Resistant Prostate Cancer; ALL: acute lymphoblastic leukemia; LLy: acute lymphoblastic lymphoma; PMF: Primary myelofibrosis; CIM: Chronic Idiopathic Myelofibrosis;post-PV MF: Post-polycythemia vera myelofibrosis; PET-MF: post-essential thrombocythemia myelofibrosis; SR-GVHD: Steroid Refractory Graft Versus Host Disease; T-ALL: Acute T-Lymphocytic Leukemia; T-LBL: T-lymphoblastic leukemia/lymphoma;T/M-MPAL: T-lymphoid/myeloid mixed phenotype acute leukemia; DLBCL: Diffuse large B-cell lymphoma; CHD: Coronary Heart Disease; HF: Heart Failure; LV: Left Ventricle; MetS: Metabolic Syndrome; NA: Not Applicable; PAH: Pulmonary Arterial Hypertension; PUFA: Omega-3 Polyunsaturated Fatty Acids; T2D: Type 2 Diabetes

**Fig. 1:**
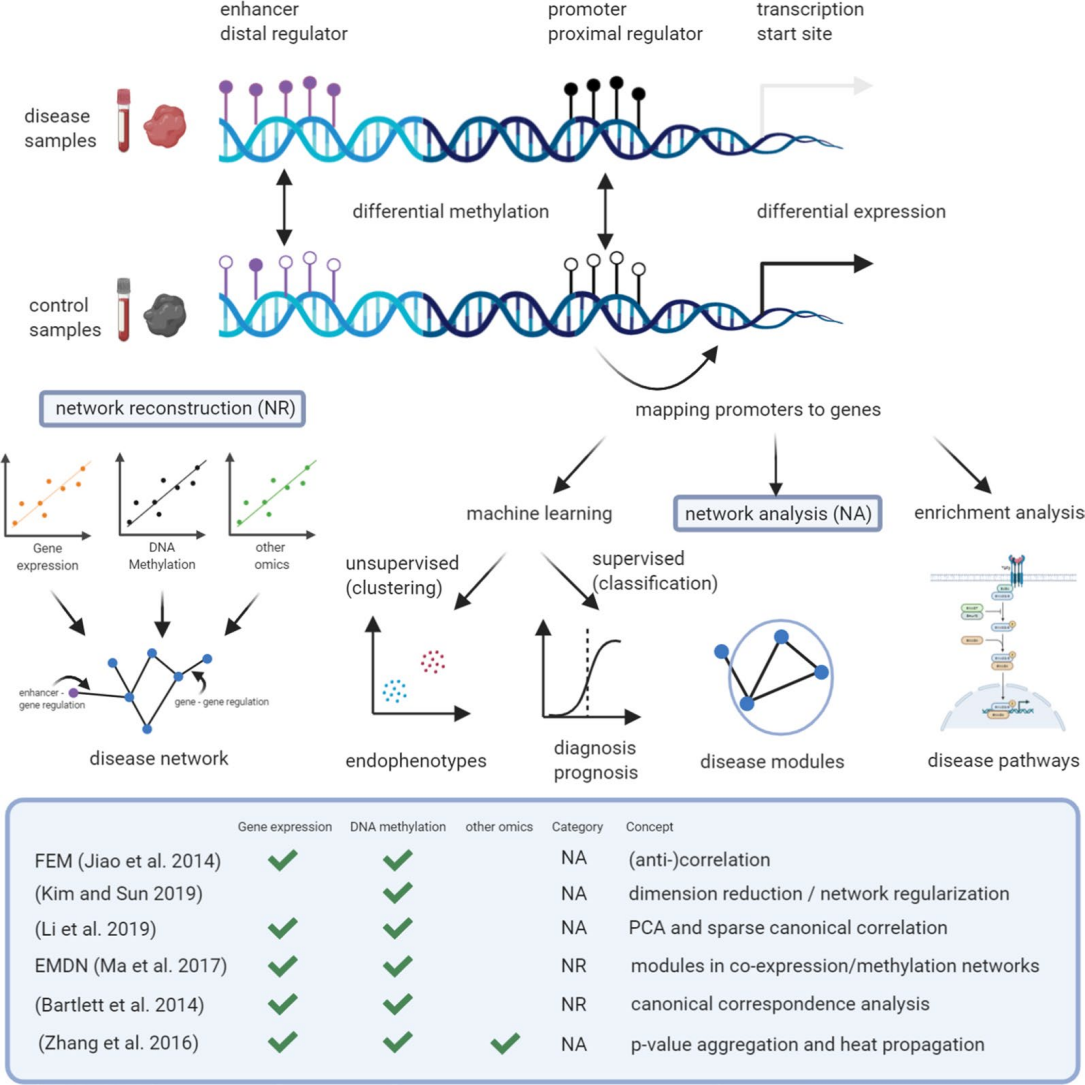
Application of NM in disease. Principles of network medicine methods (top) considering the differences of DNA methylation between disease and control samples. Network reconstruction (NR) methods build a disease network de novo while network analysis methods (NA) identify disease modules in existing networks based on prior knowledge (middle). Overview of commonly employed network medicine methods, their expected input and the concept they employ (bottom)

The historically limited success in the discovery of epigenetic biomarkers and epi-drugs using a reductionist approach calls for a paradigm shift toward network medicine (NM), which combines big data, advanced bioinformatic tools, network science, systems biology, artificial intelligence, and clinical biometric data to investigate the pathogenesis of complex diseases such as cancer and CVDs. By considering the molecular perturbations of integrated biological pathways rather than a single molecular defect ([15, 75, 92, 64, 120, 123]), NM can leverage molecular interaction networks for advancing diagnosis, prognosis, and treatment Fig. 1 [15, 75, 120, 123]. In this way, NM paves the way toward precision medicine and personalized therapy [54]. Focusing thus far mostly on genomics and transcriptomics, NM has recently been applied to the study epigenetic mechanisms such as DNA methylation changes in the pathogenesis of cancer (mostly) and CVDs Fig. 1.

The goal of this review is to present clinical applications of epigenetics in cancer and CVDs. Additionally, we discuss how NM can gradually be advanced from the bench to bedside via epigenetics. With this overview, we provide information and insights for both basic scientists and physicians who work at the interface of these new applications of clinical epigenetics and Network Medicine.

## Epi-therapy in cancer: a new frontier for the emergence of precision medicine advanced therapies

### Approved epi-treatments against hematopoietic cancers

The main classification for epigenetic mediators divides them into writers, readers, and erasers [18]. DNMTs and HATs are “writers,” as they add a methyl or acetyl group on chromatin. These residues can be removed by the “erasers” such as HDACs or KDMs. Chromatin modifications may be interpreted by “readers,” such as the CBX (chromodomain) or BRD (bromodomain) family members. Writers, readers, and erasers comprise highly studied targets, some of which have led to the identification and development of FDA-approved drugs entering into the clinics.

DNMTi and HDACi are the epi-compounds most used for treatment. DNA hypermethylation is frequently associated with cancer development, and the two DNMTis, azacitidine (Vidaza) and decitabine (Dacogen), were approved for myelodysplastic syndromes (MDS) treatment in 2004 and 2006, respectively. These hypomethylating agents are currently the first-line therapy for MDS, when stem cell transplantation is unsuitable. Administered at a daily dose of 75 mg/m^2^ for 7 days (azacitidine) and 5–20 mg/m^2^ for 5/7 days (decitabine), these treatments are more effective than previously investigated drugs [110]. However, azacitidine has some limitations, including a low response rate, short duration of action, and aggravation of thrombocytopenia, leading to its co-administration with other therapies (Table 2, NCT01488565). Azacitidine is currently also under investigation as a treatment option in solid malignancies (see Sect. 2.4 for further information).

Decitabine is well tolerated and although the most significant effects have been observed in hematologic malignancies, it also displays a good activity against solid tumors. Different Phase I/II clinical trials are currently underway, including co-administration studies with HDACi for patients with advanced solid tumors (NCT01023737, NCT02453620, NCT03925428, NCT03590054, NCT01281176) or with cisplatin for resistant ovarian cancer [87]. Decitabine has shown numerous beneficial effects in solid tumors, leading to further investigation of methylation status as a prognostic marker in solid cancers. The FDA has approved second-generation HDACi, including vorinostat (Zolinza), belinostat (Beleodaq), romidepsin (Istodax), and panobinostat (Farydak) for treatment of cutaneous T-cell lymphoma (CTCL), peripheral T-cell lymphoma (PTCL), and multiple myeloma (MM).

Mutations in R132 and R172 in isocitrate dehydrogenase 1 and 2 (IDH1/2) have been found in approximately 20% of acute myeloid leukemias (patients with a devastating prognosis [109]. These mutations produce an abnormal conversion of α-ketoglutarate (D-2-kg) in α- hydroxyglutarate (2-HG), leading to a dysfunction of the enzymes using 2-kg as a cofactor, such as TETs and KDM enzymes, associated with DNA hypermethylation, “aberrant gene expression,” increase of proliferation and cell differentiation [95]. Recently, the FDA approved the first IDH1/2 inhibitors (NCT01915498,NCT02074839) against AML. Enasidenib (IDHIFA), targeting IDH2, and ivosidenib (TIBSOVO), IDH1, are administrated orally, blocking 2-HG in the blood of AML patients with IDH mutations. The patients treated with these agents showed a complete response (CR) or CR with partial hematological recovery after 8.2 months of treatment, response was superior with ivosidenib (32.8%) superior to the enasidenib treatment (23%). Ivosidenib has been approved in 2019 for AML patients older than 75 years or for patients in whom chemotherapy cannot be used.

### Clinical breakthroughs after more than one decade of HDAC inhibitors use

Vorinostat, an HDACi used as an oral agent for CTCL since 2006 and now tested in clinical trials for solid tumors, displays low toxicity and high efficacy, making it a first-line drug for the treatment of this lymphoma. The majority of adverse effects are fatigue and gastrointestinal symptoms. Hematological abnormalities are observed only at the highest dose.

Like vorinostat, romidepsin is one of the first drugs to be used for CTCL. Romidepsin is a potent class I HDACi and its ability to inhibit class II HDACs at higher concentrations suggests that it may act as a broad-spectrum HDACi [52]. Unlike other drugs, this molecule induces a change in electrocardiographic patterns, after administration, with flattening of ST and T waves and depression of the ST segment. Six patients with mild cardiovascular disorders died after romidepsin treatment in Phase II studies. This drug also shows significant activity in patients with PTCL and in CTCL patients with stage IIB or higher disease presentations [107]. To date, romidepsin has proved unsuccessful as monotherapy in squamous cell carcinoma of the head and neck (SCCHN) [57] and in metastatic castration-resistant prostate cancer (CRPC) [94] showing limited adverse effects such as fatigue, nausea, and vomiting.

Belinostat, approved in 2014 for PTCL treatment [74], generally displays no major adverse effects, but in rare cases induces liver damage. Belinostat is currently in several clinical trials Table 1.

Panobinostat was been approved in 2015 for multiple myeloma (MM) as a second-line therapy in patients not responding to bortezomib. The limitation of panobinostat has been the low efficacy when given as a single agent; thus, it is administered in combination with bortezomib and dexamethasone. MM patients treated with panobinostat in combination with bortezomib have a significant increase in survival (NCT01023308). Additionally, when studied in a Phase III trial in non-Hodgkin lymphoma patients, panobinostat-induced adverse events in 22% of the patients with severe vomiting and diarrhea (NCT01034163), and hepatic abnormalities in chronic myeloid leukemia (CML) (NCT00449761), although it shows promising anticancer effects, alone or in combination [88].

Although HDACi are excellent candidates for integrative network analysis due to their extensively studied molecular mechanisms, no network-based approaches were reported for repurposing this class of drugs to other cancer types. However, by using a meta-analytical approach *Rafehi *et al*.* integrated ENCODE data with microarray expression profiles showing HDACi-mediated suppression of EP300 target genes, including genes implicated in diabetes mellitus [108].

### Epi-treatments being investigated for solid cancers

Mutations and overexpression of EZH2 have been associated with prostate, breast, liver, skin, lung, and gastric cancers, as well as with lymphoma and melanoma [1, 7, 8, 58, 60, 114, 129, 139]. Tazemetostat (Tazverik, Epizyme, Inc), acting as a selective competitor of S-adenosyl-L-methionine, is a first-in-class EZH2 inhibitor, administered orally against hematological and solid tumors. On June 18, 2020, the FDA granted approval to tazemetostat in adults with relapsed/ refractory (R/R) follicular lymphoma (FL) carrying an EZH2 mutation who have received at least two prior systemic therapies, or for those patients who have no alternative options. The cobas EZH2 Mutation Test (Roche Molecular Systems, Inc.) as a diagnostic test was also approved, based on two open-label, single-arm cohorts of a multicenter trial (E7438-G000-101, NCT01897571) in patients with FL after at least two prior systemic therapies.

Tazemetostat was also studied as a single agent in a Phase II clinical trial for patients with relapsed or refractory non-Hodgkin lymphoma [73], rare aggressive forms of lymphoma [85], follicular lymphoma in refractory patients in combination with atezolizumab (Tecentriq), diffuse large B-cell lymphoma (DLBCL), and is currently in the recruitment phase for prostate cancer in combination with enzalutamide or abitarerone/prednisol (NCT04179864). In combination with R-CHOP, a standard chemotherapy regimen, tazemetostat, is in ongoing studies as a first-line treatment for newly diagnosed high-risk elderly patients with DLBCL.

### Ongoing clinical trials with epi treatments against cancer

Several chromatin-acting drugs are being investigated in clinical trials; see Table 2 for a complete overview. DNMTi and HDACi administration produces a synergistic effect on methylation state, with an increase in repression of pro-oncogenic and activation of apoptotic genes [96, 106, 145]. Clinical trials in different phases testing the combined administration of both classes of drugs are yielding excellent results [19]. Although toxicity appears more frequently in older patients, azacitidine proved effective in MDS and in AML (NCT01074047), where it represents an excellent replacement therapy for patients who are not candidates for more aggressive therapies (65–74 years) and for those with cytogenetics indicating an increased risk. The HDACi entinostat, a generally well-tolerated drug, is currently in Phase II trials for co-administration with azacitidine and nivolumab in patients with metastatic non-small lung cell cancer (NCT01928576), and in melanoma and lymphoma in co-administered with pembrolizumab (NCT03179930,NCT03765229). Activity of the EZH2 inhibitor CPI-1205 in combination with the antiandrogen enzalutamide (Xtandi) is being tested in Phase I/II trials as second-line treatment of metastatic castration-resistant prostate cancer patients (NCT03480646), and showing excellent tolerance and considerable anticancer activity, prompting further studies. The DOT1L inhibitor pinometostat (EPZ-5676) is part of an ongoing Phase I/II study for the treatment of AML and MLL (mixed-lineage leukemia) (NCT03701295,NCT03724084). Tranylcypromine, an LSD1 inhibitor, is under investigation in at least 26 studies. A recent interventional study testing tranylcypromine with all-trans retinoic acid (ATRA) in AML and MDS found no serious side effects (NCT02273102), leading now to the more intensive evaluation study of its anticancer effects.

Osteosarcoma (OS) patients are generally treated with combination chemotherapy comprising cisplatin, doxorubicin, and high-dose methotrexate, with addition of ifosfamide [130]. However, a fundamental problem is the long duration of treatment, which leads to undesirable effects [118]. Several clinical trials in initial stages involve single or co-administration of epi-drugs [37]. EZH2 is a positive regulator of the growth of metastasis and prognosis of OS [38]. In January 2020, tazametostat (Tazverik) was quickly approved by the FDA for advanced metastatic and locally epithelioid sarcoma treatment in adults and pediatric patients (16 years), based on the successful results obtained in a phase 2 study (NCT02601950).

Additionally, tazametostat is in Phase 2 clinical trial against OS with mutations of EZH2, SMARCB1 and SMARCA2 (NCT03213665) in pediatric patients.

Despite the steady rise in the number of clinical studies that include epi-drugs as anticancer agents, only a few agents and combinations have proven useful for broad clinical use. Thus, a joint approach involving basic scientists and physicians for the development of clinically useful protocols is needed to improve our understanding of epigenetic mechanisms and how they can be targeted most effectively. This goal is particularly important for solid malignancies, where the use of chromatin-targeting drugs has not yet been shown to provide benefit over standard treatments. With respect to hematological malignancies, the clearly lower efficacy cannot only be attributed to limited knowledge of the oncogenic mechanism. Possible additional factors might include differences in solid tumor heterogeneity or in the 3D structures of solid malignancies where cancer cells in poorly vascularized tumor areas are difficult to reach with drugs. For all of these reasons and others to be uncovered, network medicine strategies may prove fundamental for an understanding of (epi)based treatments and for assessing responses, especially in treatment-resistant phenotypes.

## Network medicine in the clinical setting of cancer prevention and diagnosis

Integrating drug discovery and pathology with network medicine analysis provides rational and efficient approach to identify novel treatments and allows repurposing clinically approved drugs for human diseases, with implications for personalized medicine. Recent examples illustrate how network medicine has been adapted in the field of epigenetics for diagnosis, precision medicine, and patient stratification in terms of prognosis Table 3.

**Table 3:** Examples of epigenetics and network-oriented analysis in cancer and CVD susceptibility

Network-oriented analysis	Sample size	Sample source	Aim	Platform	Results	References
*CVDs*						
WGCNA, Comb-p	Discovery set 2129 women from the WHI; Replication set: 2726 subjects from the FHS	Blood	To construct a DNA methylation-oriented network and analyze possible relationships with incident CHD	HumanMethylation450 microarray	DMRs annotated to SLC9A1, SLC1A5 and TNRC6C strongly correlated with incident CHD	Westerman et al. [137]
Co-variation of enhancer activity and gene expression across study participants and GO enrichment	10 end-stage PAH patients at time of lung explant and 9 unused donor control subjects	PAECs	To construct a regulatory network based on TF-H3K27ac enhancer relationship	ChIP-Seq, Illumina RNA-Seq	A remodeling of active (H3K27ac) enhancers combined with differential transcription factors may guide a dysregulated angiogenesis and endothelial-to-mesenchymal-transition	Reyes-Palomares et al. [112]
*Cancer*						
NcADMM algorithm; LLR	562 TCGA ovarian cancer	Online data	To construct a DNA methylation-oriented network of TCGA ovarian cancer	Illumina Infinium HumanMethylation27 platform; Affymetrix HT-HGU133A platform	Identified the path associated with CCNE1, AURKA and RAB25 mediated by DNA methylation	https://doi.org/10.38/s41598-019-42010-6
GSEA, MSigDB, FEM	64endometrial cancer tissue and 23 healthy control samples	Endometrial tissue	To study new methylated biomarker test to distinguish endometrial cancers from non-cancers	Illumina Infinium HumanMethylation27K BeadChip	HAND2 methylation is a common and crucial molecular alteration in endometrial cancer that could potentially be employed as a biomarker	https://doi.org/10.1371/journal.pmed.1001551
WGCNA, GEPIA	201 patients of the TCGA prostate cancer	TCGA database	To build a network analysis correlation of RNA and DNA methylation to identify target therapy	Illumina human methylation 450 platform	This protocol has predicted the FOXD1 as predictor of poor prognosis	https://doi.org/10.2217/epi-2019-0349
WGCNA, ssGSEA, GO and KEGG pathway enrichment	1248 breast cancer patients	TCGA database	To build a DNA methylation and RNA-seq network for Brest cancer stratification patients	RNA-seq and DNA methylation datasets	Stratify breast cancer patients into low- and high-risk groups	https://doi.org/10.1186/s12967-019-2126-6
GEO, MLP	391 patients of 11 different cancer	TCGA database	To construct DNA methylation network and gene expression	Illumina human methylation 450 k BeadChip; Illumina 450 k platform	New application to classified the different cancer type based on DNA methylation levels	32384093
MCODE, K-shell method	780 samples in BRCA, 468 samples in SKCM, and 428 samples in UCEC	TCGA database	To make a DNA methylation data, mRNA expression data and clinical data network	Illumina HumanMethylation 450 K Assay	Identification of gene signatures associated with cancer prognosis	https://doi.org/10.3390/genes10080571
GREAT, LOLA, ENCODE	30 glioblastoma patients	Tissue	To study the genomic location and abundance of 5 hmC in glioblastomas to study the disease progression	IlluminaHumanMethylation450kmanifest, version 0.4.0; IlluminaHumanMethylation450kanno.ilmn12.hg19, version 0.2.1	Identification of a global loss of 5 hmC in glioblastoma compared with healthy prefrontal cortex tissues	https://doi.org/10.1038/ncomms13177
Affymetrix Genome Wide SNP Arrays v6; WGS; ENCODE; HotNet	200 AML	Blood	To make a DNA-methylation network with RNA and microRNA to investigate the AML pathogenesis, classification, and risk stratification	Affymetrix U133 Plus 2 platform; Illumina Infinium HumanMethylation450 BeadChip; Affymetrix SNP Array 6.0; Illumina HiSeq 2000; Illumina GAIIX	Identification of pathway that stratified the AML patients	https://doi.org/10.1056/NEJMoa1301689
WGS; ATAC-seq; WGBS; ENCODE	410 TCGA samples from 23 cancer types	TCGA database	To build DNA regulatory elements and gene promoter network for future integrative gene regulatory analyses	Illumina MiSeq Sequencer;	Identification of transcription factors and enhancers driving molecular subtypes of cancer associated with clinical prognosis	10.1126/science.aav1898
GREAT; GSEA; ATAC-seq	Mammary tumors from mouse models and human patients	Tissue	To create a network-chromatin accessibility and transcriptional profiling during mammary development to identify factors that mediate cancer cell state interconversions	Illumina HiSeq 2500	Identification of SOX10 that binds the genes that regulate neural crest	https://doi.org/10.1016/j.ccell.2018.08.001
SNF; GO and KEGG pathway enrichment	185pancreatic cancers	Tissue	To build a mRNAs, miRNAs and DNA methylation network for pancreatic cancer patient stratification	Informatic platform	Identification various signaling cascade associated with different tumor subtype	https://doi.org/10.1038/s41598-020-58290-2
scRRBS; NONCODE, ENCODE	26 single cells isolated from a 51-year-old male HCC patient	Tissue	To use a DNA methylation, RNA-seq and CNV network in HCC single cell	scTrio-seq; Illumina HiSeq2000 or HiSeq 2500 Sequencer	Identification of new approach to study the heterogeneity and complexity of cell populations in development and cancer interrogating in the same time the genome, methylome, and transcriptome	https://doi.org/10.1038/cr.2016.23
MACs2; ENCODE; GREAT	CML cells	Cell culture	To build an ATAC-seq and RNA-seq network in single cells	Illumina HiSeq 4000; NextSeq; qRT-PCR	Correlation between GATA and CD24 that induce a high genetic and epigenetic variability, and resistance to imatinib mesylate treatment	28118844
WGBS; WGS; MSigDb	100 castration-resistant prostate metastases	Tissue	Introduction of whole-genome, whole-methylome and whole-transcriptome sequencing network in metastatic cancer to study the regulatory role of methylation	ChIP–seq; RNA-seq; Illumina Novaseq 6000	Identification of a novel epigenomic subtype associated with hypermethylation and somatic mutations in TET2, DNMT3B, IDH1, and BRAF	https://doi.org/10.1038/s41588-020-0648-8

Abbreviations: CHD: Coronary Heart Disease; CVDs: Cardiovascular Diseases; EF: Ejection Fraction; FHS: Framingham Heart Study; GEO: Gene Expression Omnibus; GSNCA: Gene Set Net Correlations Analysis; HF: Heart Failure; HTx: Heart Transplantation; miRNAs: microRNAs; PAECs: Pulmonary Arterial Endothelial Cells; PAH: Pulmonary Arterial Hypertension; PCA: Principle Component Analysis; PPIs: Protein–Protein Interactions; RAC1: Ras-related C3 Botulinum Toxin Substrate 1; RT-PCR: Real Time-Polymerase Chain Reaction; SLC1A5: Sodium-Dependent Neutral Amino Acid Transporter; SLC9A1: Na + /H + Antiporter; T2D: Type 2 Diabetes; TF: Transcriptional Factor; TNRC6C: Trinucleotide Repeat Containing Adaptor 6C; WGCNA: Weighted Gene Co-Expression Network; WHI: Women’s Health Initiative;LLR:L1-regularized logistic regression; MLP:multilayer perceptron; BRCA:breast invasive carcinoma; SKCM:skin cutaneous melanoma; UCEC:uterine corpus endometrial carcinoma; SNF:Similarity Network Fusion; HCC:human hepatocellular carcinoma; CNV:Copy Number Variation Analysis; CML: chronic myeloid leukemia; WM164: metastatic human melanoma cell line established from a metastatic site in a 22-year-old male with stage IV superficial spreading melanoma; tCNNS:Convolutional Neural Network for drugs in SMILES format; GDSC:Genomics of Drugs Sensitivity in Cancer; DeepDR:deepdrug response; CCLE: Cancer Cell Line Encyclopedia; DNN:deep neural network; NcADMM: nonconvex alternating direction method of multipliers; WGS: Whole Genome Sequencing; WGBS: whole-genome bisulfite sequencing; GSEA: Gene Set Enrichment Analysis; MSigDB: Molecular Signatures Database; GEPIA: Gene Expression Profiling Interactive Analysis;GO: Gene Ontology; KEGG: Kyoto Encyclopedia of Genes and Genomes; MLP: multilayer perceptron; MCODE: Molecular Complex Detection; ENCODE: Encyclopedia of DNA Elements; NONCODE: non-coding RNAs database

### Network medicine and biomarkers in cancer prevention, diagnosis, management and prognosis

DNA methylation is the most widely investigated epigenetic mechanism in network analysis Fig. 1. Changes in DNA methylation in promoter or other regulatory regions can reveal associations to cancer development and drug resistance [2, 26, 47, 82] and allow predictions with high accuracy (reviewed in [111]). For instance, Capper et al. [23] trained a random forest classifier to distinguish between tumor entities of the central nervous system. The resulting epigenetic signatures typically consist of many features with unknown mechanistic explanation. Moreover, given that features tend to be highly correlated, they can often be replaced in a signature without a loss of accuracy. A consequence of this often-observed lack of robustness [133] is that we cannot distinguish between surrogates and features causally involved in the disease. For functional enrichment analysis, features (e.g., CpGs or regulatory regions such as promoters, repressors, or enhancers) are typically mapped to the closest gene using tools such as GREAT [89]. Such a gene-mapping is also necessary to leverage existing networks such as protein–protein interaction (PPI) or gene-regulatory networks in epigenomics data analysis, where the putative target gene with the closest transcription start site is considered. West et al. showed that such a mapping strategy can be used to extract subnetworks of cancer-related differentially methylated genes [135]. Subsequently, Jiao et al. proposed the functional epigenetic modules (FEM) method [67], which revealed HAND2 as a methylation hotspot in the endometrium and indicator of drug response in progesterone treatment [69]. Kim and Sun [71] showed that PPI networks are beneficial in network-regularized feature selection after dimension reduction. Li et al. used principal component analysis for feature reduction and sparse canonical correlation analysis to infer edge weights for gene pairs. A network-based pathway-extending approach using DNA methylation and gene expression data identified altered pathways [77]. Rather than relying on a PPI network as prior information, disease-specific regulatory networks can be inferred directly from epigenomic data. With their epigenetic module based on differential networks (EMDN) algorithm, Ma et al. used The Cancer Genome Atlas Program (TCGA) data to construct both a co-expression and a co-methylation network [84]. Bartlett et al. [11] used canonical correspondence analysis of methylation profiles to score pairs of interacting genes to construct a gene co-regulatory network. Edges significantly associated with survival were used to extract subnetworks that exhibited functional enrichment relevant to the cancer context investigated here. Recently, a deep neural network (DNN) algorithm applied to DNA methylation data from 7339 patients across 18 different TCGA tumors classified the origin of cancer [143]. Using crosstalk between genetic modules, Cui et al. [35] created a co-methylation network based on DNA methylation data. The K-shell algorithm, applied to three types of cancer, invasive breast carcinoma, skin cutaneous melanoma, and uterine corpus endometrial carcinoma, identified the main genes in the modules that are predictive of prognosis and classification. In invasive breast carcinoma, this network method identified ten genes responsible for metastasis and tumor progression. *RCHY1* was found at the junction of two modules, closely related to the histone lysine demethylase KDM1B, which plays a key role in methylation and silencing. In addition to the methylation state, modification of the methylene group on DNA can be associated with cancer diagnosis. The conversion of 5-methylcytosine (5mC) to 5-hydroxymethylcytosine (5hmC) was studied in 30 glioblastomas using the OxyBS algorithm with an enrichment analysis via Genomic Regions Enrichment of Annotations Tool (GREAT). The results implicate the depletion of 5hmC in various cancer types, which is associated with an increase in proliferation markers [68]. Despite these promising early results in the network-based integration of DNA methylation in oncology, clinical translation of epigenomics-based network medicine methods is still lacking. In particular, the integration of epigenomics data in a multi-omics context remains challenging. A weighted correlation network analysis (WGCNA) of 201 patients in a TCGA prostate cancer dataset revealed hypermethylation of FOXD1 as an unfavorable marker for survival [141]. The combination of mRNA expression and DNA methylation datasets in WGCNA and downstream gene ontology (GO) enrichment using single sample gene set enrichment analysis (ssGSEA) yielded a 13-gene epigenetic signature associated with survival of breast cancer patients. This panel of genes considered upregulation of known cancer-related pathways (e.g., mTOR signaling) to distinguish high- from low-risk cancer cases [9]. The genomes of 200 clinically annotated adult cases of de novo AML were studied using whole-genome or whole-exome sequencing as well as RNA-seq, microRNA-seq, and DNA methylation profiling. A potential driver mutation in DNA methylation and RNA-seq (e.g., in *DNMT3A*, *NPM1*, *CEPBA*, *IDH1/2*, and *RUNX1*) promoting AML pathogenesis in individual patients was found in all AML samples (Cancer Genome Atlas Research et al. [22]. The tool SWItchMiner (SWIM) was applied to RNA-sequencing data from TCGA [90] to characterize disease etiologies and to identify potential therapeutic targets [103]. In glioblastoma multiforme (GBM), SWIM revealed new insights into the molecular mechanism determining the stem-like phenotype of glioblastoma cells [49]. Here SWIM implicated FOSL1 as a putative master regulator of a core of four master neurodevelopmental transcription factors (i.e., SOX2, SALL2, OLIG2, POU3F2), whose induction was demonstrated to be sufficient to reprogram fully differentiated glioblastoma cells into stem-like cells [125]. More recently, SWIM was used in conjunction with network analysis to enhance disease module discovery [104]. Corces et al. [34] generated high-quality ATAC-seq data for 410 TCGA samples and identified cancer- and tissue-specific DNA regulatory elements in 23 cancer types. These chromatin accessibility profiles allowed the classification of cancer subtypes with a newly recognized prognostic importance. A gene co-expression network associated with ATAC-seq analysis was built by Dravis et al. [39] to analyze breast cancer biomarkers. Here, SOX10 was identified as the major transcription factor that binds to genes responsible for neural activity.

### Network medicine in cancer patient stratification using single-cell analyses

Network medicine has enabled the identification of molecular markers defining cancer subtypes [81]. Sinkala et al. stratified 185 patients with pancreatic cancer into two groups via proteomics data. Next, they built patient-similarity networks for each of the molecular data types in TCGA which they then integrated into a joint network using similarity network fusion (SNF) [121]. SNF clustered the patients into two subtypes based on their joint molecular profiles. In a subsequent step, the K-nearest neighbor (KNN) algorithm and support vector machines (SVM) were used to identify representative biomarkers for these clusters in individual data types.

While it has not yet entered clinical practice, single-cell epigenetics is an exciting new frontier that allows for studying small and previously unrecognized cell populations such as stem cells Table 4 [56, 122], preimplantation embryos [144], and the heterogeneity of subpopulations of human tissues [53, 83]. Single-cell epigenetics also has the potential to be applied in the diagnosis and prognosis in cancer treatment, where identifying the mutational profile might potentially impact disease evolution and response to treatment. Several sequencing protocols are developed in Table 4, including the single-cell bisulfite sequencing (scBS-seq) method to determine the DNA methylome of cell populations [122]. Recent single-cell approaches combine the methylome with RNA transcription profiling [63]. Moreover, single-cell chromatin accessibility profiling with scATAC-seq has provided the opportunity to recognize different populations within the same tumor and to discriminate cancer cell populations and their heterogeneity [21]. Recently, scTRIO-seq, which allows parallel profiling of genomic, transcriptomic, and DNA methylation in single cells, has led to the identification of two different hepatocellular carcinoma (HCC) subpopulations in the same patient. Based on the DNA and transcriptome of 25 HCC cells, *Yu Hou *et al. found two distinct populations differing in DNA copy numbers, DNA methylation, or RNA expression levels, of which the smaller tumors expressed more invasive cell markers [61]. scATAC-seq, used to identify co-varying transcription factor-cell surface marker pairs, was combined with scRNA-seq for cell surface marker expression to detect efficiently CD24-labeled chronic leukemia cells as co-varying with chromatin accessibility changes linked to GATA transcription factors [79]. A positive correlation was found between CD24 and GATA levels, characterized by high genetic and epigenetic variability, conferring resistance to imatinib mesylate treatment.

**Table 4:** Epi-single-cells technique developed for cancer studies

Technique	Function and description
*DNA methylation in single cells*	
*scBS-seq*	The first technique developed for methylome analysis. It degrades 90% of DNA and does not distinguish 5mC from 5hmC
*scRRBS-seq*	Technique for methylome analysis.Compared to scBS-seq, reduces DNA loss
*MID-RRBS*	RRBS in a microfluidic support, obtaining an efficient bisulfite conversion with high DNA recovery
*PBAT*	Technique for methylome analysis.It does not involve DNA fragmentation. This goes to reduce the loss
*MRSE*	Using the restriction enzymes to recognize methylation sites coupled with PCR amplification, reduced costs, and reaction times
*RGM*	It uses a fluorescence reporter to analyze dynamic changes in the state of endogenous methylation, discriminating completely the methylated genome from the single allele
*scCGI-seq*	Endonuclease-based method, which improves coverage and efficiency
*scTRIO-seq*	scTrio-seq that can be used to simultaneously analyze the genomic copy-number variations (CNVs), DNA methylome, and transcriptome of an individual mammalian cell
*scMAB-seq*	Methylome technique that use enzymes for DNA conversion. Distinguishes 5mC and 5hmC from 5fC (5-formylcytosine) and 5-caC (5-carboxycytosine)
*scAba-seq*	Methylome technique used to specifically detect 5hmC by restriction enzyme. It has low efficiency
*DNA methylation analysis with gene expression*	
*scMT-seq; scM&T-seq*	Methylation associated with transcription
*scTRIO-seq*	Gene promoter and hypo- and hyper-methylation study
*scNMT-seq*	Nucleosome, methylation, and transcription
*Single-cell histone modification sequencing*	
*scChip-seq*	Method used to analyze protein interactions with DNA
*scChil-seq; scCUT&Tag-seq; scChIL-seq; scChIC-seq*	Technologies that isolate the DNA sequences attached to histones with specific marks or transcription factors
*scATAC-seq*	Technique that gives the possibility to improve the data obtained with DNA and RNA sequencing, with the information of chromatin accessibility
*scCOOL-seq*	Detection of chromatin status/nucleosome localization, DNA methylation, copy number variation, and ploidy
*ISH-PLA*	Technique for RNA regulation study, gives information on histone modification by imaging, cell phenotype, and cell–cell interaction

### Network medicine for identification of cancer treatment response vs resistance

In the last 50 years, the identification of epigenetic molecular alterations acting as drivers of cancer development and progression have transformed the clinical practice of oncology from non-specific cancer cell elimination with nonspecific chemotherapies to a more cancer-selective approach that leverages molecular profiles [12]. Predicting drug resistance, and therefore, modifying treatment according to the phenotypic response of the tumor, remains a major challenge. Several resources, such as the Genome of Drug Sensitivity in Cancer (GDSC) database, provide information required to check tumor resistance to specific drugs (https://www.cancerrxgene.org/). Technologies for large-scale genomic and epigenomic profiling allowed the full (bulk) characterization of different tumors, revealing that they share similar driver mutations and enzymatic alterations [117]. For example, upregulation of SETDB1 and SETDB2 was found in resistant cells exhibiting a loss of H3K4me3 and H3K27me3 and an increase in H3K9me3 [3]. HDAC deregulation produced an aggressive phenotype in lung cancer cells resistant to doxorubicin [40]. The resistance to epigenetic therapies for a subpopulation of leukemia stem cells found in AML models [50] was not driven by genetic evolution, but was due to epigenetic plasticity [72]. Epigenetic modifications to centrosome proteins led to tumor development and drug resistance. The Manteia [127] gene ontology analysis data system reported a correlation between lysine acetyltransferase (KAT) 2A/B alteration and serine/threonine-protein kinase PLK4 overexpression, producing resistance to tamoxifen and trastuzumab [51]. ChIP-seq and DNA methylation profiles were used to study epigenetic profiles in drug-resistant melanoma, lung, and colon cancers. Thirteen genes associated with the interferon (IFN) pathway were found to be regulated by histone modifications, including the histone methyltransferase EZH2 [3]. Recent trials found that the response to a targeted drug depends on the anatomical cancer type. For example, BRAF-mutated melanoma, NSCLC, and hairy cell leukemia satisfactorily responded to vandetanib, a drug that targets the BRAF V600E mutation, while BRAF-mutated CRC does not [117]. Falcone et al. used a network approach based on the SWIM algorithm [44] to compare pairs of BRAF-mutated cancers and found a great number of switch genes suggesting that the cancer network of each tumor is different. A number of putative targetable kinases encoded by switch genes were reported in lung adenocarcinoma and thyroid cancer, while only one was found in colorectal cancer. Interestingly, the results were in accordance with clinical trial data showing a better response rate to vemurafenib in papillary thyroid cancer patients (overall response rate of 38.5%) than in colorectal cancer patients (ORR 4.8%) [44]. These results highlight the limit of the reductionist approach where typically one gene is implicated with one disease, as this strategy does not reflect the complexity of complex diseases such as cancer and CVDs in which many potential disease genes interact.

Yildirim et al. [138] built a drug–target network, in which each drug was connected to its target proteins, and two complementary projections of it: a drug network, in which nodes are drugs and two drugs are connected to each other if they share the same targets, and a target–protein network, in which the nodes are proteins and they are connected together if are targeted by the same drug. The authors observed that new drugs tend to target the already validated target proteins and that many clinically FDA-approved drugs do not target known disease-associated genes but are rather palliative drugs. A strategy aimed to increase the efficacy and to reduce the risk of adverse effects of monotherapy, is the therapy with a combination of multiple drugs. By analyzing the network-based relationship between drug pairs, their targets, and the proteins in the disease modules Cheng et al. revealed that drugs used in combination with synergistic effect have their drug–target modules overlapping with disease modules but well separated to each other in the human interactome [30, 31]. Additionally, Tang et al. [126] developed a logic-based network pharmacology modeling approach, called TIMMA (Target Inhibition interaction using Minimization and Maximization Averaging), based on the integration of drug–target interaction profiles and single-drug sensitivities, to predict synergistic drug combinations. By applying TIMMA to the single-drug sensitivity profiles and to the kinome-wide drug–target interaction of 41 kinase inhibitors in MDA-MB-231 cell line, the authors found a synergistic target interaction between inhibition of Aurora B, a key regulator of mitosis, and ZAK, a key regulator of p38 MAPK pathway, findings also confirmed in vitro.

Twin Convolutional Neural Network for Drugs in SMILES format (tCNNS) creates a drug correlation network using the SMILES chemical representation and information on the cell phenotype [80]. Pharmacological dose–response prediction can be also obtained by DeepDR based on mutation data from TCGA, a pre-trained expression encoder, and a predictor network for drug response. From 9.059 tumor samples, DeepDR predicted cancer drug resistance and personalized therapy [32]. For resistant tumor treatment, one of the main unanswered questions is whether the cell acquires mutations during development, or whether there is a small group of cells within the same population that survives therapy. Single-cell analysis specifically allows for studying the complexity of a population [70]; however, the use of NM in epigenetics for the prediction of response versus treatment is only its inception [104].

### Epi-therapy in cancer: can Network Medicine help physicians at the bedside?

Integrating network medicine with anticancer drug discovery programs would make a significant contribution to improving patient health. Recently, whole-genome bisulfite sequencing (WGBS) integrated with whole-genome sequencing (WGS) and RNA-seq was applied to 100 metastatic prostate biopsies to sequence the methylome whole genome, and transcriptome, respectively [142]. In 22% of resistant metastases, DNA methylation analysis identified an epigenetic subtype associated with hypermethylation and mutation of TET2, DNMT3B, IDH1, and BRAD, and regions where methylation is associated with AR, MYC, and ERG expression. NM has also been used in this context to predict potential implications of therapeutics. The administration of DNMTi, such as azacitidine or decitabine, which are used for myelodysplastic syndrome (MDS) treatment, may be advantageous in resistant metastases associated with hypermethylated gene promoters. Pursuing a strategy that allows for the identification of tumor subtype targets or a specific resistance mechanism may make it possible to repurpose drugs used in the treatment of other diseases.

## Epigenetics and risk stratification of CVDs: network medicine is on the horizon

### Metabolic syndrome, diabetes, and foodome project

A notable expansion of the prevalence of metabolic syndrome (MS) has impact on the global risk of CVDs, in particular in aging Western world populations, which are prone to a high-fat diet and a sedentary lifestyle [42]. MS arises from complex crosstalk between genetic and epigenetic factors underlying obesity, in particular central abdominal obesity, that can strongly increase the risk for coronary heart disease (CHD), type 2 diabetes mellitus (T2D), and cancer [42, 64, 98, 99]. In clinical practice, a panel of five well established cardiometabolic risk factors including abdominal obesity, increased triglycerides, decreased HDL cholesterol, hypertension, and hyperglycemia is assessed by physicians, and at least three of these must be present [42]. However, in precision medicine and personalized therapy, individual DNA methylation profiles might not only represent a promising tool for the prediction, diagnosis, and prognosis of obesity and MS, but also for improving treatments to lose body weight [113]. DNA methylation changes are key molecular drivers underlying the risk of MS upon detrimental exposures (e.g., nutritional patterns), especially during early development as well as during postnatal life, offering a possible framework by which to explain the “missing heritability” of MS [36, 113]. Recently, a targeted DNA pyrosequencing and logistic regression analysis has revealed a significant positive relationship between DNA methylation levels at specific CpG islands in promoters of the *PPARα* and *LPL* genes and serum triglyceride levels (TG) in visceral adipose tissue samples from 53 MS patients in comparison with 55 healthy subjects [24]. In addition, a negative association linked methylation levels of the tumor necrosis factor gene with TG, glucose levels, HDL-c, and blood pressure suggesting a relevant factor potentially involved in preventing MS occurrence [24].

A study using the Illumina Methylation EPIC Beadchip on 1999 blood samples isolated in the Coronary Artery Risk Development in Young Adults (CARDIA) study has unveiled a strong positive association between accelerated epigenetic aging and both the MS severity score and the risk of developing MS, after adjusting for known risk factors [97]. This observation led to suggest that pharmacological and non-pharmacological interventions targeting the epigenetic aging process at the molecular level may potentially prevent MS.

Network analysis would be of paramount importance to understand the underlying molecular determinants and pathogenic processes that guide in the development of new predictive biomarkers and prevention tools. Thus far, network analysis has already been applied to separate MS components, mainly in T2D [48, 98, 99, 119] and obese-T2D patients [62], and to construct gene regulatory networks, these analyses, however, did not consider epigenetic factors.

The epigenetic clock and nutritional epigenomics may contribute to aging as well as age-related diseases [4]. Thus, mapping food-related chemical profiles (diet) and molecular pathways is the main goal of the network-based “Foodome” project (https://www.barabasilab.com/projects). Foodome emphasizes the use of digital eating patterns (“barcode”) to define the personalized exposures to nutritional–chemical compounds [10]. Merging the barcode with genetic profiles and clinical data might fuel innovative platforms that can provide insights into the molecular basis for the “diet-mutation-disease risk” axis useful for precision medicine [10]. Since epigenetics plays a important role in the diet–genome crosstalk underlying vascular damage and CV risk, even during fetal development [36, 98, 99, 101], the “individual foodome” could be integrated in longitudinal cohort studies investigating how epigenetic features change over time and in response to different nutritional exposures leading to the onset of CVDs.

### Coronary heart disease (CHD)

Endothelial and systemic inflammation plays a relevant role in disease pathophysiology, and changes in DNA methylation levels of targeted genes may be causal or predispose to disease, contributing to destabilization and rupture of atherosclerotic plaques leading to acute cardiovascular events [64, 65, 101, 115]. A recent study has emphasized the possible role of a DNA methylation-based risk score in optimizing the traditional predictors of CVD risk [136]. In addition, there is a possible correlation between blood-based methylation levels in the unique CGI regulating the human leukocyte antigen-G (HLA-G) gene, which encodes for an anti-inflammatory molecule with immunomodulatory properties, and cardiac computed tomography angiography (CCTA) features in patients with obstructive in comparison with non-obstructive CHD [116]. Hypomethylation of a specific fragment of CGI-associated HLA-G gene positively correlated with coronary calcium score and was predictive for disease severity suggesting that methylation might not only have a critical role in disease severity but also a role as noninvasive biomarker(s) improving the prognostic value of CCTA [116]. The WGCNA and Comb-p algorithms have been applied to the identification of blood-based differentially methylated regions (DMRs) and disease modules associated with incident CHD events in two independent cohorts (discovery set: 2129 women, replication set: 2726 subjects) [137]. This study has identified two modules highly enriched for development and immune-related processes. In addition, a multivariate analysis has revealed a positive correlation with BMI, highly sensitive C reactive protein (hsCRP), and TG [137]. Three DMRs annotated to the sodium/hydrogen exchanger 1 (SLC9A1), solute carrier family 1 neutral amino acid transporter member 5 (SLC1A5), and trinucleotide repeat containing adaptor 6C (TNRC6C) genes significantly replicated across the two cohorts, providing possible useful predictive biomarkers [137]. However, the possible cause–effect relationship between methylation changes in these genes and CV risk needs to be determined.

### Pulmonary arterial hypertension (PAH)

PAH is a rare and incurable disease characterized by vasoconstriction and consequent elevated pulmonary artery pressure owing to three main endophenotypes, endothelial dysfunction, cell proliferation/migration, and inflammation triggered by an interplay between genetic and epigenetic risk factors with exposure to detrimental environmental stimuli [25, 86, 100]. Data regarding the potential clinical relevance of differential epigenetic factors in PAH, mainly changes in DNA methylation, have been increasing in the past few years, and network-oriented approaches are helping to prioritize novel candidate genes and drug targets [100]. Recently, an integrated regulatory network has been constructed by integrating chromatin with transcriptomic and interaction profiling in pulmonary arterial endothelial cells (PAECs) obtained from end-stage PAH patients at the time of lung explant and control subjects [112]. As a result, an in-depth remodeling of active enhancers marked by H3K27ac and regulated by specific transcription factors may trigger perturbation of angiogenesis and endothelial-to-mesenchymal transition processes in PAECs in response to specific growth factor signals, as experimentally confirmed for target genes such as nitric oxide synthase 3 (NOS3) [112]. However, further studies will investigate a possible correlation between key gene regulatory networks and underlying PAH severity or responsiveness to vasodilatory therapy.

### Heart failure (HF) and heart transplantation (HTx)

Heart failure (HF) affects approximately 20% of general population and contributes to 11% of deaths with an estimated incidence that will rise by 25% over the next 15 years [16]. HF can develop asymptomatically for years, and once diagnosed, the effectiveness of most drug therapy interventions are modest. Nonetheless, the recent tremendous impact, both of SGLT2 inhibitors and angiotensin–neprilysin inhibition, will likely reduce HF mortality [66, 132]. However, the development of novel early biomarkers useful for stratification and/or as prognostic markers is a priority in the clinical setting of HF. Based on the ejection fraction (EF) value, we can classify three clinical phenotypes: HF with reduced EF (HFrEF), HF with mid-range EF (HFmrEF), and HF with preserved EF (HFpEF) [98]. An epigenetic-based phenotype mapping strategy of HFrEF, HFmrEF, and HFpEF patients seems to be a possible option to identify noninvasive biomarkers discriminating differential HF subgroups and/or novel drug targets to test in clinical trials aimed at establishing specific therapeutic strategies for each phenotypic profile [99]. However, the availability of cardiac biopsy from living patients and controls in humans is scarce; thus, most pathogenic studies are based on animal models. A pioneer multi-omics study integrated the methylome and transcriptome of left-ventricular biopsies and whole peripheral blood samples of 41 patients with HFmrEF caused by dilated cardiomyopathy and compared to 31 patients who underwent routine left-heart myocardial biopsy after receiving transplantation, as a control group. The promoter DNA hypomethylation of the natriuretic peptide A and B (NPPA and NPPB) genes regulates overexpression of these genes providing a novel putative class of HF biomarkers easily detectable in peripheral blood [91].

Graft surveillance after heart transplantation is a challenge in the management of transplanted patients, and current guidelines indicate that invasive endomyocardial biopsy is the gold standard to diagnose and monitor organ rejection. Ideally, graft rejection may be diagnosed and predicted by noninvasive biomarkers present in the peripheral blood or other biological fluids [99]. Specifically, an increasing interest in circulating epigenetic molecules is providing novel findings that may aid physicians in a more accurate risk stratification [99]. DNA methylation is a pivotal contributor to a balanced immune response toward the graft, due to regulation by both the innate and adaptive immune systems, with primarily T cells as the key players of alloreactivity and targets for immunosuppressive drugs [27, 28]. Interestingly, *FOXP3* gene expression was significantly higher in biopsy samples of rejectors collected before rejection in comparison with non-rejectors, and showed the tendency to predict rejection events [20]. Longitudinal studies could evaluate the possible role of this biomarker in the clinical setting of HTx.

## Repurposed drugs and epitherapy in CVDs: network medicine can improve the current reductionist approach to reach the goal of personalized treatments

As the discovery and development of novel drugs is a highly expensive time-consuming process, the repurposing of “old” drugs to treat CVDs is increasingly becoming an attractive goal. The repurposing of both metformin and statins has been widely evaluated in large controlled clinical trials for the prevention and treatment of CHD, PAH, HF, and complications after HTx Table 2. Metformin proved in 1994, is the first-line oral drug for treatment of T2D and obesity (www.ncbi.nlm.nih.gov/books/NBK409379). Metformin has glucose-lowering effects by acting on several molecular pathways and also represents an agonist of the SIRT1 enzyme [98, 99]. Additionally, the first statin (lovastatin) received FDA approval in 1987 and now 6 statins, including simvastatin and pravastatin (semi-synthetic), as well as fluvastatin, atorvastatin, rosuvastatin, and pitavastatin (synthetic), have been introduced to the market providing first-line of oral drugs prescribed for dyslipidemias and prevention of atherosclerotic plaque development [41]. Basically, statins block the 3-hydroxy-3-methylglutaryl coenzyme A (HMG-CoA) reductase enzyme leading to lipid-lowering effects and also showing epigenetic interference (HDACi). The cardio-protective and anti-inflammatory effects of metformin and statins have also been widely demonstrated in patients affected by CVDs [98, 99, 101]; however, large controlled trials are needed to establish whether these beneficial effects are conferred by the glucose and lipid-lowering effects, by interference with specific epigenetic-sensitive pathways, such as inflammatory pathways or by both. Another example of drug repurposing includes tocilizumab (Actemra), a monoclonal anti-IL-6 antibody approved by FDA in 2010 for treatment of rheumatoid arthritis. In addition, there are a few examples of properly defined epi-drugs under clinical evaluation for treatment of CVDs. One of the most promising is apabetalone (RVX-208), inhibiting selectively the bromodomain and extra-terminal domain (BET) family of proteins binding to acetyl groups in order to normalize acetylation imbalance underlying cardiac dysfunction in T2D, CHD, PAH, and HF Table 2.

### Ongoing and completed clinical trials

In contrast to cancer patients, epitherapy has not yet reached clinical practice in the management of CVDs. However, exploring the website http://clinicaltrial.gov/, we retrieved a large number both of ongoing observational and interventional clinical trials (Phase 3 and 4) of these agents in CVDs Table 2 demonstrating the great interest in translating epigenetic findings from the bench to bedside to treat patients affected by CVDs.

#### Diabetes and CHD

A completed double-blinded, randomized, placebo-controlled Phase 3 trial (NCT02773927) compared the effect of metformin/agave inulin in comparison with agave inulin on adiponectin levels in patients with metabolic syndrome by grouping patients into in four clusters: group A, metformin plus agave inulin; group B, metformin plus placebo of agave inulin; group C, agave inulin plus placebo of metformin; group D, placebo of agave inulin plus placebo of metformin. To date, there are no published results from this trial. Metformin is also currently under investigation in a Phase 2, observational, prospective clinical trial (NCT01884051) enrolling 1.899 participants, in which the primary endpoints are measures of insulin resistance, urinary, and plasma oxidant stress markers, right ventricle lipid content, oxidative metabolism, and drug safety. As secondary endpoints, the quantification of lung metabolism, [18F]‐fluorodeoxyglucose uptake, blood-based expression of the bone morphogenic protein receptor (BMPR) 2, right ventricle ejection fraction and volumes using magnetic resonance imaging, insulin resistance, and 6-min walking distance (6MWD) is evaluated. Additionally, the preliminary results from an ongoing Phase III trial (NCT02586155) have demonstrated possible efficacy in preventing myocardial damage in high-risk T2D-CHD patients under high-intensity statin therapy combined with RVX 208 (daily dose 100 mg capsule plus atorvastatin and rosuvastatin).

#### Pulmonary arterial hypertension

At the molecular level, trimetazidine can switch the metabolic cellular state from beta-oxidation toward glucose oxidation by inhibiting synthesis or carnitine-facilitated transport of fatty acids so that cardiomyocytes can obtain more energy [131]. Recently, it has been reported that trimetazidine may exert its cardio-protective role in women by affecting the DNA methylation profile of the cyclin dependent kinase inhibitor 2b (CDKN2B) gene [27, 28]. Trimetazidine is being studied in a randomized controlled trial (NCT03273387) which aims to determine if 3 months of treatment (35 mg twice a day) combined with standard therapy can alter right ventricle function in PAH patients. The primary endpoint will be a change in right ventricular function, as quantified by cardiac magnetic resonance imaging (MRI). Secondary endpoints will contain modifications in cardiac fibrosis quantified by T1 cardiac MRI mapping, functional class, and plasma levels of lactate dehydrogenase (LDH). However, tocilizumab is being investigated in the “*Therapeutic Open‐Label Study of Tocilizumab in the Treatment of Pulmonary Arterial Hypertension study*” (NCT02676947), a single‐arm study aimed at investigating whether treatment with tocilizumab in a dose of 8 mg/kg monthly for 6 months may alter pulmonary vascular resistance in PAH patients [59]. The primary endpoints will be a change in pulmonary vascular resistance (PVR) and safety, with secondary endpoints including 6MWD, N-terminal pro-B-type natriuretic peptide (NT‐pro‐BNP), symptom burden, and quality of life. This trial is still ongoing Apabetalone is under investigation in a single‐arm trial in a two‐center study enrolling PAH patients combining the standard therapy with 100 mg of apabetalone twice a day for 16 weeks (NCT03655704).

#### Heart failure (HF) and heart transplantation (HTx)

An interesting example of drug repurposing arises from the randomized, double-blind, placebo-controlled cross-over intervention DoPING-HFpEF trial (EU Clinical Trial Register: 2018-002170-52; NTR registration: NL7830), which will evaluate the possible cardio-protective effects of trimetazidine in patients affected by HFpEF [131]. A large randomized, double-blind study enrolling 6.975 HF patients with monitored EF provided evidence for which assumption of 1 g per day of long-chain omega-3 polyunsaturated fatty acids (PUFA) is associated with a small reduction (9%) in mortality and admission to the hospital for CV events in HF patients [128]. Dietary supplementation of PUFA may aid in normalizing circulating lipid levels, exerting beneficial systemic anti-inflammatory effects, preventing cardiac injury by affecting blood-based global DNA methylation levels [98, 101].

### Which additional clinical benefits would the network medicine approach add?

The interactome may reveal differential pathogenic molecular drivers in each patient that are, in part, responsible for current limitations of the one-size-fits-all approach ([15, 64, 76, 86]). Indeed, CVD patients treated with optimized standard-of-care regimens can still show high residual morbidity and mortality risk [5]). Thus, interactome-based selection before enrolling subjects in a clinical trial may aid in selecting more homogeneous study populations in which to test a specific drug therapy. NM approaches would potentially resolve this heterogeneity by combining the interactome with biochemical and molecular assays and clinical information, potentially providing noninvasive biomarkers as well as drug targets ([14, 64, 98, 100, 120]). A network-oriented paradigm of CVD pathogenesis would have the power of repurposing existing FDA-approved compounds by in silico prediction of targeted disease modules to speed up the discovery of putative novel personalized treatments [29]. Moreover, repurposing bioinformatic tools could offer new molecular mechanisms of existing drugs. As an example, the seed connector algorithm (SCA) has been applied to GWAS-derived CHD seed proteins and has suggested that the neuropilin-1 (NRP1) would be potentially a novel candidate disease gene [134]. Since NRP1 is the target of the anti-angiogenic agent pegaptanib, which is indicated for the treatment of neovascular age-related macular degeneration, the SCA algorithm approach suggested its possible repurposing for atherosclerotic diseases [134]. In addition, the use of network-based proximity measures would allow to quantify the relationship between CVD modules and drug targets helping to chart novel associations and discriminate if a candidate repurposed drug may be therapeutically effective or lead to unwanted side effects [55]. Similarly, another proximity score has highlighted a relevant role for dysregulation of the immune system in MS development, suggesting the repurposing of ibrutinib, a BTK inhibitor prescribed for hematological malignancies, to counteract the inflammatory state [93].

## Concluding remarks network medicine’s role in clinical medicine, challenges and a pathway forward to the new of precision medicine

The management of hematological malignancies has already seen a major benefit from epigenetics with nine epi-drugs currently approved by the FDA. Although clinical epigenetics is far less advanced in CVDs, large clinical trials show the promising results with respect to the effectiveness of drug repositioning and epitherapy in the treatment of MS, T2D, CHD, PAH, HF, and HTx. Therefore, in areas relevant to the cause of death, such as CVDs, there need to be more expeditious ways to repurpose drugs as we look toward the era of precision medicine.

The reasons for this gap may also be due to the different procedures for approval of clinical studies in cancer versus CVD. Several epigenome-wide association studies have revealed molecular pathways involved in the pathogenesis of cancer and CVDs that may offer robust biomarkers for precision medicine and personalized therapy. Novel technological developments as well as the application of NM may change our view of the role and analysis of epigenome deregulation in disease. The development and use of bioinformatics (and tools of artificial intelligence) hints at a deep change when dealing with human health, disease identification and handling.

NM applications may improve our mechanistic understanding of tumorigenesis and the dynamics of driver and contributing (epi)mutations within the 3D structure of the cell and of tissues. The indication that a cytosine is first methylated and then hydroxymethylated suggests that the order of (some) epigenome changes should be possibly integrated mining the epigenome landscape in health and disease. Furthermore, different chromatin complexes (such as the readers and some erasers) use metabolic co-factors in their reactions, suggesting that, among the regulatory networks, the availability of those cofactors might, in turn, also regulate the prioritization of events. Indeed, the integration of genome and epigenome information together with the metabolic status of the cells might, in principle, be a necessary step forward to stratify better epi-biomarkers and epi-targets in disease.

Studying the role of spatiotemporal regulation of chromatin with integrative network approaches may represent the next step forward for personalized medicine, allowing drug-based fine-tuning of the epigenome. While a small number of computational tools already leverage epigenetic profiling data in a network context, most existing approaches are limited to promoter methylation, neglecting the influence of regulatory regions distal to promoters. Recently, the EpiRegio database established a link between *cis*- or *trans*-regulatory elements and their target genes [13]. In combination with disease-specific molecular profiling data, network enrichment [78] or disease module discovery [33] can be employed on such networks to reveal more distal and complex regulatory interactions for developing clinically relevant insights into epigenetic mechanisms in diseases in the future. However, these approaches are not yet well understood by physicians. Learning workshops on this groundbreaking field are necessary for the fields of cardiology and oncology. Interpreting the developments of epigenome deregulation to the clinic may also need a further understanding of the use of epi-drugs. For example, physicians should be aware that the reduced locus-selective specificity may lead to (epi)genome undesired effects. Novel drug discovery approaches targeting DNA mutations might be a more focused solution, as indicated for the EZH2 inhibitors discussed in the previous sections. In addition, methods such as “*the proteolysis targeting chimera (PROTAC)*” might produce specific drugs, perhaps able to (re)modulate the function of non-enzymatic chromatin complexes.

A future challenge will, thus, be to integrate such notions in a network context together with other modalities such as transcriptomics, proteomics, and miRNA expression as well as with clinical information. We expect that integrative network analysis will reveal regulatory patterns that can be exploited for diagnosis, prognosis, and treatment selection. We need to encourage the scientific integration of basic scientists and physicians in the field of clinical epigenetics. A relevant role is also played by innovative technology assets applied to the clinical setting and management of patient. None of NM approaches has yet reached clinical application, but some have been validated with excellent results in *ex-vivo* patients Table 5. Therefore, the final step will be to evaluate the potential of novel NM platforms in large clinical trials to test their reliability value in diagnosing, prognosticate, and treating cancer and CVDs, as two of the major causes of death worldwide.

**Table 5:** Network medicine approach in cancer and CVDs

Network medicine	System validation	Data obtained	
FEM	Endometrial tumor samples and 23 healthy controls	DNA methylation of HAND2 gene in 90% of tumors	Jiao et al. [67], Jones et al. [69]
OxyBS	Fresh frozen glioblastoma specimens from 30 subjects diagnosed between 2004 and 2012	5-Hydroxymethylcytosine patterns are strongly related to transcription, localizes to disease-critical genes and are associated with patient prognosis	Johnson et al. [68]
WGBS + WGS	Biopsy 100 castration-resistant prostate metastases	Discovery of association between hypermethylation and somatic mutations in TET2, DNMT3B, IDH1 and BRAF in epigenomic subtype	Zhao et al. [142]
WGCNA	Blood samples isolated from 2627 subjects enrolled in FHS cohort	Differences in circulating DNA methylation signatures located in regulatory regions of SLC9A1, SLC1A5, TNRC6C genes may be useful biomarkers to predict incident CHD	Westerman et al. [137]

Abbreviations: FEM: Functional Epigenetic Modules; OxyBS: oxidative bisulfite and bisulfite; WGBS: whole-genome bisulfite sequencing; WGS: whole-genome sequencing; WGCNA: weighted correlation network analysis; FHS: Framingham Heart Study

## Data Availability

Not applicable.

## Abbreviations

2-HG: α-Hydroxyglutarate
2-kg: α-Ketoglutarate
5hmC: 5-Hydroxymethylcytosine
5mC: 5-Methylcytosine
AML: Acute myeloid leukemia
BET: Bromodomain and extra-terminal domain
BMI: Body mass index
BMPR: Bone morphogenic protein receptor
BTK: Bruton’s tyrosine kinase
CCTA: Cardiac computed tomography angiography
CDKN2B: Cyclin-dependent kinase inhibitor 2b
CEPBA: CCAAT enhancer binding protein alpha
CHD: Coronary heart disease
CML: Chronic myeloid leukemia
CpG: Cytosine-phosphate-guanine dinucleotide
CR: Complete response
CRPC: Castration-resistant prostate cancer
CTCL: Cutaneous T-cell lymphoma
CVD: Cardiovascular disease
DLBCL: Diffuse large B-cell lymphoma
EMDN: Epigenetic module based on differential networks
EZH2: Enhancer of zeste 2 polycomb repressive complex 2 subunit
FDA: Food and drug administration
FEM: Finite element method
FL: Follicular lymphoma
FOXD1: Forkhead Box D1
FOXP3: Forkhead box P3
GBM: Glioblastoma multiforme
GDSC: Genome of drug sensitivity in cancer
GO: Gene ontology
GREAT: Genomic regions enrichment of annotations tool
DNMT: DNA methyl transferase
DNN: Deep neural network
H3K4me3: Histone 3 lysine 4 methylation
H3K9me3: Histone 3 lysine 9 methylation
H3K27ac: Histone 3 lysine 27 acetylation
HAND2: Heart and neural crest derivatives expressed 2
HAT: Histone acetyl transferase
HCC: Hepatocellular carcinoma
HDAC: Histone deacetylases
HDL: High-density lipoprotein
HF: Heart failure
HLA-G: Human leukocyte antigen-G
HMG-CoA: 3-Hydroxy-3-methylglutaryl coenzyme A
hsCRP: High sensitive C reactive protein
HTx: Heart transplantation
IDH: Isocitrate dehydrogenase
IFN: Interferon
KAT: Lysine acetyl transferase
KDM: Lysine demethylase
KNN: K-nearest neighbor
LDH: Lactate dehydrogenase
LPL: Lipoprotein lipase
MAPK: Mitogen-activated protein kinase
MDS: Myelodysplastic syndrome
MLL: Mixed-lineage leukemia
MM: Multiple myeloma
MS: Metabolic syndrome
NM: Network medicine
NOS3: Nitric oxide synthase 3
NPPA: Natriuretic peptide A
NPPB: Natriuretic peptide B
NRP1: Neuropilin-1
NT‐pro‐BNP: N-terminal pro-B-type natriuretic peptide
PAECs: Pulmonary arterial endothelial cells
PAH: Pulmonary arterial hypertension
PLK4: Polo-like kinase 4
PPARα: Peroxisome proliferator-activated receptor alpha
POU3F2: POU class 3 homeobox 2
PPIs: Protein–protein interactions
PTCL: Peripheral T-cell lymphoma
PUFA: Long-chain omega-3 polyunsaturated fatty acids
OLIG2: Oligodendrocyte transcription factor
OS: Osteosarcoma
OxyBS: Oxidative bisulfite and bisulfite
R/R: Relapsed/refractory
RUNX1: RUNX family transcription factor 1
SALL2: Spalt-like transcription factor 2
SCCHN: Squamous cell carcinoma of the head and neck
SETDB1/2: SET domain bifurcated histone lysine methyltransferase 1/2
SGLT2: Sodium/glucose cotransporter 2
SIRT1: Silent information regulator 1
SLC9A1: Sodium/hydrogen exchanger 1
SLC1A5: Solute carrier family 1 neutral amino acid transporter member 5
SMARCA2: SWI/SNF related, matrix associated, actin-dependent regulator of chromatin, subfamily A member 2
SMARCB1: SWI/SNF-related matrix-associated actin-dependent regulator of chromatin subfamily B member 1
SOX10: SRY-Box transcription factor 10
ssGSEA: Single sample gene set enrichment analysis
SVM: Support vector machines
SWIM: SWItchMiner
T2D: Type 2 diabetes
TCGA: The Cancer Genome Atlas
tCNNS: Twin convolutional neural network for drugs in SMILES format
TET: Ten–eleven translocation
TIMMA: Target inhibition interaction using minimization and maximization averaging
TG: Triglyceride
TNF: Tumor necrosis factor
TNRC6C: Trinucleotide repeat containing adaptor 6C
WGBS: Whole-genome bisulfite sequencing
WGCNA: Weighted correlation network analysis
WGS: Whole-genome sequencing
